# PRegnancy Outcomes after a Maternity Intervention for Stressful EmotionS (PROMISES): study protocol for a randomised controlled trial

**DOI:** 10.1186/1745-6215-12-157

**Published:** 2011-06-20

**Authors:** Judith L Meijer, Claudi LH Bockting, Chantal Beijers, Tjitte Verbeek, A Dennis Stant, Johan Ormel, Ronald P Stolk, Peter de Jonge, Mariëlle G van Pampus, Huibert Burger

**Affiliations:** 1 Department of Epidemiology, University Medical Centre Groningen, Hanzeplein 1, 9700 RB, Groningen, The Netherlands; 2 Department of Clinical and Developmental Psychology, University of Groningen, Grote Kruisstraat 2/1, 9712 TS Groningen, The Netherlands; 3 Interdisciplinary Centre for Psychiatric Epidemiology, University Medical Centre Groningen, Hanzeplein 1, 9700 RB, Groningen, The Netherlands; 4 Department of Obstetrics and Gynaecology, Onze Lieve Vrouwe Gasthuis, P.O. Box 95500, 1090 HM Amsterdam, The Netherlands

## Abstract

**Background:**

There is ample evidence from observational prospective studies that maternal depression or anxiety during pregnancy is a risk factor for adverse psychosocial outcomes in the offspring. However, to date no previous study has demonstrated that treatment of depressive or anxious symptoms in pregnancy actually could prevent psychosocial problems in children. Preventing psychosocial problems in children will eventually bring down the huge public health burden of mental disease. The main objective of this study is to assess the effects of cognitive behavioural therapy in pregnant women with symptoms of anxiety or depression on the child's development as well as behavioural and emotional problems. In addition, we aim to study its effects on the child's development, maternal mental health, and neonatal outcomes, as well as the cost-effectiveness of cognitive behavioural therapy relative to usual care.

**Methods/design:**

We will include 300 women with at least moderate levels of anxiety or depression at the end of the first trimester of pregnancy. By including 300 women we will be able to demonstrate effect sizes of 0.35 or over on the total problems scale of the child behavioural checklist 1.5-5 with alpha 5% and power (1-beta) 80%.

Women in the intervention arm are offered 10-14 individual cognitive behavioural therapy sessions, 6-10 sessions during pregnancy and 4-8 sessions after delivery (once a week). Women in the control group receive care as usual.

Primary outcome is behavioural/emotional problems at 1.5 years of age as assessed by the total problems scale of the child behaviour checklist 1.5 - 5 years.

Secondary outcomes will be mental, psychomotor and behavioural development of the child at age 18 months according to the Bayley scales, maternal anxiety and depression during pregnancy and postpartum, and neonatal outcomes such as birth weight, gestational age and Apgar score, health care consumption and general health status (economic evaluation).

**Trial Registration:**

Netherlands Trial Register (NTR): NTR2242

## Background

The burden of mental disorders is huge and at least comparable to the burden caused by many severe physical diseases. In the WHO Global Burden of Disease project it was estimated that 50% of all daily adjusted life years (DALY's) in the 15-44 years old are due to nine psychiatry-related conditions [1]. Depressive disorders are projected to rank second on a list of 15 major diseases in terms of burden of disease in 2030 [2]. In addition, a substantial part of the costs are caused by new cases, which accounts for 39.2% of the costs at population level [3]. Therefore, prevention of mental disorders is essential.

Maternal anxiety or depression during pregnancy is an important and potentially modifiable risk factor for cognitive, behavioural and emotional problems in her children [4-8]. Around 10-20% of all women are suffering from depression or anxiety during pregnancy [9-12]. The magnitude of the effects of maternal anxiety or depression on the child's psychosocial problems is considerable: it is estimated that up to 22% of the variance in behavioural problems is linked with prenatal anxiety, stress or depression [6]. The adverse effects seem to be lasting. For example, antenatal anxiety of the mother was related to behavioural or emotional problems of 4 year old children, independent of the mother's postnatal depression or anxiety [4], and higher anxiety levels of the mothers early in pregnancy were related to an increase in ADHD and other externalizing problems in their 8-9 year old children [13].

There are several ways in which depression or anxiety during pregnancy could have an adverse effect on the offspring. These ways can be divided in direct and indirect ways.

In the direct way, depression activates the maternal stress system leading to elevated cortisol levels, which might influence the development of the foetus' brain by passing the placenta.

An indirect adverse effect might be another mechanism, because women who suffer from antenatal depression have the tendency to take less good care of themselves (e.g. neglecting personal hygiene, sleeping problems, disturbed drinking and smoking habits, denying prenatal care). These consequences might all influence the development of the foetus [14-17].

Another indirect way in which depression might influence the offspring is when the antenatal depression remains after delivery and turns into a postnatal depression. In this way, mother-child attachment might be endangered, because the mother has a reduced ability to respond to the child. Children from depressed mothers have a higher risk on insecure attachment, which in turn is associated with cognitive, behavioural and emotional problems [1,18-20].

Finally, the association between antenatal depression and adverse outcomes in the offspring might be explained by genetic predisposition in both mother and child.

The effectiveness of psychological therapy in the treatment of both anxiety and depression has been shown in the past 50 years, especially for cognitive behavioural therapy (CBT) [21-25]. Although guidelines state that medication is an alternative effective treatment, the safety of antidepressants during pregnancy remains insecure [26].

Still, it is too early to implement CBT for depressed or anxious women to prevent psychosocial problems in the offspring. This is because in the development of such a preventive strategy, demonstration of the causality and size of the effect of the reduction of symptoms of depression and anxiety on child outcomes is a crucial step, a step that has not been taken to date. This knowledge gap will be filled by the results of the present experimental study.

Whatever the actual mechanisms involved are, there is presently convincing evidence that children whose mothers suffered from anxiety or depression during pregnancy constitute a high risk group for behavioural and emotional problems. On population level, substantial total mental health gains may be accomplished when depressed or anxious women are adequately treated during their pregnancy, even if the effect size of the treatment is relatively small.

We will perform a randomized controlled trial (RCT) in pregnant women with symptoms of depression or anxiety to study the effect of CBT as compared to care as usual (CAU) on the offspring's behavioural and emotional problems.

In the CBT arm, we expect more beneficial neonatal outcomes, in particular higher birth weight and less prematurity, which are risk factors for adverse cognitive and behavioural outcomes themselves [8]. We also anticipate reduced smoking and less drinking, with many physical and mental health benefits for the child as a result [17]. Since prenatal depression has shown to be related to postnatal depression, we hypothesize that our intervention will also counter postnatal depression, which in turn will benefit the mother - child attachment [27].

Finally, but not unimportantly, the reduction of symptoms of anxiety or depression during pregnancy and the early postnatal period is valuable in itself. It may further provide for a safer approach to reducing symptoms in pregnancy than antidepressant medication [15]

To date, no such study has been performed.

## Methods/Design

### Objective

The aim of this study is to examine the effect of cognitive behavioural therapy (CBT) in women with at least moderate symptoms of anxiety and/or depression at the end of the first trimester of pregnancy, on the extent of total behavioural and emotional problems in their children at 1.5 years of age, as compared with care as usual (CAU).

### Setting & Design

The source population will consist of all pregnant women in the Netherlands in the first trimester of their pregnancy. Women are recruited in obstetric care. In addition, we will invite pregnant women through advertisements in regional and national media targeted at pregnant women.

Women will be screened on anxiety and depression symptoms. Women with at least moderate symptoms of anxiety and/or depression at the end of the first trimester of pregnancy are either randomized to the intervention group in which they receive 10-14 sessions of CBT, or to the control group in which they receive care as usual.

**Figure 1:**
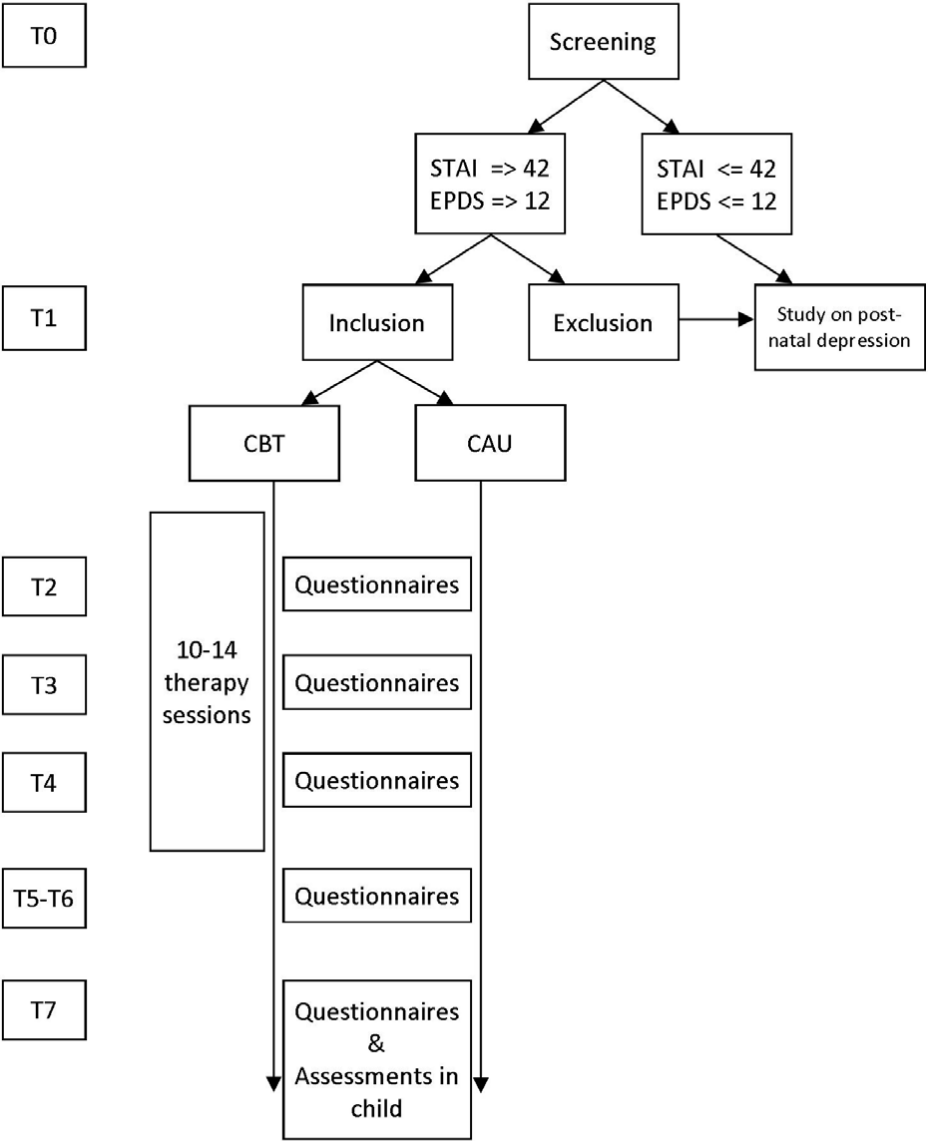
**Flow diagram**.

Figure 1 shows the exact design of the study.

### Study outcome measures

The primary outcome in this project is the total emotional and behavioural problems score of the child according to the Child Behaviour Check List 1.5 - 5 (CBCL 1.5-5) at 18 months of age.

Secondary outcomes are the child's mental and psychomotor development at 18 months of age, the change in depressive and anxious symptoms in the mother, obstetric variables such as birth weight, gestational age and Apgar score, the socio-demographic and lifestyle factors, such as alcohol use, smoking and education, and cost-effectiveness of the therapy.

### Sample size

Studies on the prevention of mental disorders tend to suffer from problems of insufficient statistical power [28]. In the current study we aim to get around this problem by using a continuous primary outcome measure and by including a high risk group (selective prevention).

We decided that effect sizes of 0.35 (midpoint of small - medium effect size) or over on the total problems scale of CBCL 1.5-5 are to be detected. With alpha 5% and power (1-beta) 80%, we have to include 260 participants in our analyses. To account for some drop out we aim at 300 women entering the trial. If 50% eventually meets all criteria and gives informed consent, 600 screen-positives must be identified. The 50% rate is based on studies with psychological interventions during pregnancy aimed at reducing the occurrence of postnatal depression [27]. Given the figures in the literature [11,29] we can expect around 10% screen-positives on either the anxiety or depression screener. With an estimated 50% comorbidity between anxiety and depression this means that approximately 15% will be eligible for the randomisation. Therefore, 4000 need to be screened. Assuming a response rate of 75% [30] this implies that 5333 must be offered screening. To be on the safe side, we aim at screening 6000 women. If necessary for the recruitment of 300 women, the total number of screens may be increased.

### Inclusion

Women in obstetric care in the Netherlands with a significant level of anxiety (6 item STAI > = 42) or at least moderate depressive symptoms (EPDS > = 12) in their first trimester are invited to participate in the trial.

### Exclusion

Women fulfilling one or more of the following criteria will be excluded from participation:

1. Multiple pregnancy. We decided to exclude women with multiple pregnancy as they have a markedly increased obstetric risk; inclusion will threaten the homogeneity of the study population and thereby decrease the sensitivity to detect effects.

2. High suicidal risk according to the suicidality subscale score on the MINI International Neuropsychiatric Interview (MINI, defined as a positive response on the question on concrete suicide plans)

3. Presently receiving psychotherapy

4. Substantial physical disease or illegal substance abuse

5. No mastery of the Dutch language

6. Having a psychiatric history on bipolar disorder, psychoses and manic disorder

7. History of in vitro fertilization

### Assessments

Women will be asked to fill in questionnaires until their child is 1.5 years. This will be done at 8 occasions: the screener at baseline (T0), the additional baseline information (T1), and follow-up questionnaires at 24 and 36 weeks of gestation (T3 and T4), at 6 weeks postpartum (T5), 6 months postpartum (T6), 12 months postpartum (T7) and 18 months postpartum (T8).

At all time points, levels of anxiety and depression are monitored by the 6-item State Trait Anxiety Inventory (STAI) and the Edinburgh Postnatal Depression Scale (EPDS). As to be found in table 1, all other questionnaires can be filled in once or at several time points.

**Table 1:** Assessments per measurement

	T0	T1	T2	T3	T4	T5	T6	T7
**Depression (EPDS)**	X	X	X	X	X	X	X	X

**Anxiety (STAI)**	X	X	X	X	X	X	X	X

**Personality NEO-FFI**		X		X		X		

**Life events before pregnancy (NLEQ)**		X						

**Life events during pregnancy**		X		X		X		

**Perceived social support (SSQ)**		X						

**Coping styles (UCL)**		X	X	X	X			

**Attitudes (DAS)**		X	X	X	X			

**Maternal attachment (ECR)**		X					X	

**Quality of life (EQ-5D)**		X	X	X	X	X	X	

**Sociodemographic & -economic factors**		X						

**Lifestyle**		X		X		X		

**Breastfeeding**						X		

**General health**		X						

**Health care consumption**		X			X	X	X	

**Previous pregnancies**		X						

**Suicidality (MINI)**		X						

**Clinical Diagnostic Interview (SCID-II)**		X						

**Child Behaviour (CBCL)**								X

**Child development (BSID-II)**								X

For anxiety, we use the Dutch version of the 6-item STAI. This self report questionnaire is as valid as the full 20-item version and has frequently been used to measure antenatal anxiety [29]. For the screening on depression we use the EPDS, which has 10 items [31]. This is the most frequently used self report depression screener in the postnatal period as well as during pregnancy and has been found particularly valid during pregnancy because this scale omits somatic symptoms [30].

The following information will be obtained from participants. The exact time of administration can be found in table 1.

• Life events before pregnancy will be assessed at baseline, using the Negative Life Events Questionnaire [NLEQ, [32]].

• Perceived social support will be measured according to the 9-item Social Support Questionnaire (SSQ)-short form [33].

• General health, socioeconomic position, ethnicity, smoking behavior, alcohol use, psychiatric history (whether the participant has had depression and/or anxiety symptoms before, whether she was treated for this and whether she is presently in treatment for these symptoms) will be assessed. Socioeconomic position will be assessed using five indicators: family income, educational level (father and mother), and occupational level (father and mother). This questionnaire is based on a questionnaire used in the Utrecht Health Project (Dutch acronym LRGP: Leidsche Rijn Gezondheids Project, http://www.zorggegevens.nl/zorg/eerstelijnszorg/leidsche-rijn-gezondheidsproject/). General health status will also be taken into account according to the EQ-5D [34].

• Personality will be assessed using the NEO Five Factor Inventory (NEO-FFI). The NEO-FFI is a shortened version of the NEO-PR-I [35] and covers the Big Five of personality (neuroticism, extraversion, openness, altruism and conscientiousness). These aspects each contain 6 subscales. The NEO-FFI contains 60 questions, 2 on each subscale. The present study will add 4 full subscales to the short version; two subscales of neuroticism, one of extraversion and one of conscientiousness. This is because we expect them to have the strongest association with persistence of depression and/or anxiety. The NEO-FFI is translated and validated in Dutch [36].

• Information on previous pregnancies, family size and composition, pregnancy related life events and on reactions on becoming a parent will be administered using questionnaires from the ALSPACstudy (http://www.bristol.ac.uk/alspac).

• Suicide risk will be measured using six screening questions from the MINI International Neuropsychiatric Interview [MINI, [37]].

• Maternal attachment style will be measured according to the ECR [38], which has been translated and validated for the Netherlands by Conradi et al. [39].

• Health care consumption will be assessed based on the TIC-P [40]. This instrument allows reliable recall over the past 6 months [41].

• Coping style will be assessed using the Utrechtse Coping Lijst, the UCL [42].

• A Dutch version of the Dysfunctional Attitude Scale (DAS) will be used to measure cognitions and attitudes [43].

• Obstetric variables such as gestational age, birth weight, Apgar score, complications such as (pre)eclampsia or HELLP, which will be obtained from midwifes. Women will be asked to give consent for this.

• Finally, we will use the SCID-I to screen for a possible clinical depressive or anxiety disorder [44]. The SCID-I is the only questionnaire used that has to be taken in a personal interview.

Besides questionnaires for the mother during her pregnancy and the first 1.5 years postpartum, there will be assessments in the child at 1.5 years of age.

One of the assessments is the Bayley Scale of Infant Development (BSID-II) [45]. This is a formal neuropsychological tool to assess the developmental level of a child between 1 and 42 months. It is individually administered by one of the researchers and consists of 3 subscales: cognitive development (mental development index), gross and fine motor development and the behavioural rating scale. This tool is widely used in both research and clinical settings and is considered the best and most applied method for the assessment of the child's development to date [46]. Importantly, the instrument has shown to be sensitive. In the context of our proposal, maternal anxiety in pregnancy explained as much as 11% of the variance in the Bayley scores in a study among two year old toddlers by LaPlante et al. [47].

The second assessment is the Child Behaviour Check List 1.5 - 5 (CBCL 1.5-5) including the Caregiver-Teacher Report form (C-TRF) and the Language Development Survey (LDS) [48]. This well established, reliable and valid scale designed for parents and caregivers comprises seven syndrome scales: emotionally reactive, anxious depressed, somatic complaints, withdrawn, sleep problems, attention problems and aggressive problems. In addition, it contains scales for internalizing, externalizing and total problems. Symptom scores may further be related to formal DSM-diagnostic criteria. The LDS provides a screen for delays in vocabulary and word combinations.

For the assessment of psychopathology in preschool children it is essential to obtain information from different sources [49]. Therefore we decided to include the C-TRF for the caregivers of the children other than their parents. Parents will be asked to hand these lists to the actual caregivers of their children, e.g. grandparents, baby-sitters, kindergarten-coaches, et cetera. Relevant in this respect, a review by Skovgaard [46] underlined the significance of both the developmental aspects (e.g. as measured with the BSID II) and the infant caregiver relation in the assessment of children 0-3 years of age.

The CBCL has been used successfully in several studies, amongst others on externalizing problems [50]. It has been translated and standardized for use in around 60 countries, including the Netherlands. The CBCL 1.5-5 is considered a sensitive instrument also deployed in current intervention studies [51,52]

Also, mother-child interaction will be measured by taping them for 15 minutes on video and scoring them afterwards on interaction points.

All assessors of both handling questionnaires filled in by the mother and measuring the children at age 1,5 are blinded for treatment assignment.

### Additional baseline data

Women agreeing to participate will be asked to provide additional baseline data at T1, as to find in table 1.

About half of these questionnaires are sent to the participants in print, the other half can be answered online. All follow-up questionnaires are available online.

After providing baseline data both in print and online, women are telephoned for the Structured Clinical Interview for DSM-II Disorders (SCID-VI). The SCID-II will allow us to study treatment effects additionally according to diagnostic categories rather than symptom levels.

### Randomisation

Right after the SCID-II interview, women are randomised 1:1 to either CBT or CAU.

We will create randomisation lists, stratified for parity and socio-economic position, with randomly permuted blocks of random size.

Women randomized to the CAU arm will be informed about being at risk of depression or anxiety disorder by the researchers and will be advised to contact their GP. A close record will be kept of all care provided in the CAU arm.

### CBT Intervention

The intervention consists of 10-14 individual sessions: 6-10 sessions during pregnancy and 4-8 sessions after delivery (once a week). The CBT will be conducted by registered psychologists, specialized in conducting CBT.

CBT posits that an individual's biased information processing leads to maladaptive feelings and behaviours which can culminate in psychological distress and eventually in psychiatric disorders. The main focus of the proposed intervention is targeted on identifying and changing dysfunctional cognitions and schemata (attitudes) specifically for pregnant depressed and anxious patients. In CBT, the Socratic dialog is used aiming to change these dysfunctional cognitions and attitudes permanently. CBT may therefore result in long term protection against psychosocial problems. It is therefore not surprising that cognitive therapy during the acute phase of depression also appears to be effective in reducing subsequent recurrence rates [25].

The first session will focus on the rationale CBT, i.e. the influence of (irrational or dysfunctional) cognitions and attitudes on feelings and behaviours. Additionally, goal setting will be initiated. These therapy goals will be unique for each patient. The subsequent sessions will be targeted at identifying and amending irrational cognitions and attitudes related to pregnancy, delivery, concerns about the (unborn) child and the future family situation. Each session will address specific pregnancy-related cognitions. Additionally, patients will be taught how dysfunctional cognitions and attitudes affect adversely feelings and behaviours.

These dysfunctional cognitions and attitudes will be challenged and replaced by functional cognitions and attitudes. After each session, patients will be given home work. For example, patients will be asked to register negative experiences, and accompanying cognitions, feelings and behaviours. Finally, in the last two to four sessions, the newly learned cognitions and attitudes will be consolidated.

### Data analysis

If necessary, skewed continuous variables will be transformed to normality prior to the analyses. The primary outcome, i.e. the CBCL scores at month 18, will be compared between the treatment arms using the unpaired t-test. This test will also be used for detecting differences in the Bayley scores by month 18 and the obstetric variables measured at birth. The latter group of variables will be tested using the Chi2 test if categorical. Differences in attachment style at month 12 will be analyzed using analysis of covariance with the baseline variable as a covariate. Continuous outcomes that were measured more than twice (e.g. EPDS and STAI) will be analyzed as dependent variables using linear mixed models for fixed and random effects. These models are superior for the analysis of longitudinally correlated data and can optimally deal with missing values [53]. A mixed model ascribes a unique intercept and slope estimate to each individual, while making use of information across individuals for predicting these quantities. In these analyses, a treatment*time variable indicating the effect of the intervention will be included as an independent variable. If despite randomisation important baseline differences exist in prognostically important variables such as the extent of social support or history of life events, they will be adjusted for. Additional analyses will be conducted to demonstrate mediation of the effect of CBT on the child's outcomes by maternal symptom level, alcohol or nicotine consumption in pregnancy, medication use or neonatal outcomes.

The analyses will primarily be carried out according to the intention-to-treat (ITT) principle, i.e. the participants will be analyzed according to their randomized allocation, regardless of the actual CBT undergone, or time in study after baseline. Aside from the optimal validity of ITT analyses, they quantify the effects on the outcome measures that would be obtained in practice. The magnitude of the effect measured in an ITT analysis incorporates the effects caused by non-adherence to CBT, behavioural changes, et cetera. Secondary analyses will be of the 'per protocol' type meaning that they will be restricted to those women that had all of the CBT sessions.

Considering specific target populations, there is evidence that the socio-economically deprived may have more benefit from treatment of depression during pregnancy [15]. Therefore, subgroup analyses will be undertaken according to socio-economic position. Subgroup analyses will also be undertaken according to parity.

Differences in effect of CBT between subgroups will be statistically evaluated by testing treatment by subgroup interaction terms. Effect parameters will be supplied with a 95% confidence interval.

### Economic evaluation

An economic evaluation will be conducted alongside the trial to assess the cost-effectiveness of CBT compared to care as usual in the current study population. Information on costs and health outcomes will be prospectively collected during 24 months (starting at baseline until 18 months after birth) for both mother and child. Two complementary economic analyses will be conducted. The primary outcome measure of the planned cost-effectiveness analysis is the total emotional and behavioural problems score of the child according to the CBCL at 18 months of age.

In the additionally planned cost-utility analysis, QALYs (Quality Adjusted Life Years) will be used as the primary outcome measure. The study will be performed from a societal perspective. Medical costs that will be assessed include costs related to CBT, contacts with healthcare professionals, and medication use. Outside the healthcare sector, costs of informal care and productivity losses will be taken into account. Unit prices will largely be based on Dutch standard prices in order to facilitate comparisons with other economic evaluations. Cost-effectiveness acceptability curves will be used to inform decision-makers on the probability that the studied intervention is cost-effective.

This study protocol was approved by the medical ethical committee of the University Medical Centre Groningen.

## Abbreviations

ADHD: Attention Deficit Hyperactivity Disorder; ALSPAC: Avon Longitudinal Study Parents And Children; BSID II: Bayley Scale of Infant Development; CAU: Care As Usual; CBCL: Child Behavioural CheckList; CBT: Cognitive Behavioural Therapy; C-TRF: Caregiver-Teacher Report Form; DALY: Daily Adjusted Life Years; DAS: Dysfunctional Attitudes Scale; DSM: Diagnostic and Statistical manual of Mental disorders; ECR: Experiences in Close Relationships scale; EPDS: Edinburgh Postnatal Depression Scale; EQ-5D: EuroQol; HELLP: Hemolytic anemia, Elevated Liver enzymes and Low Platelet count; ITT: Intention To Treat; LDS: Language Development Survey; MINI: Mini International Neuropsychiatric Interview; NEO-FFI: NEO Five Factor Inventory; NEO-PI-R: Revised NEO Personality Inventory; NLEQ: Negative Life Events Questionnaire; PROMISES: PRegnancy Outcomes after Maternity Intervention for Stressful EmotionS; QALY: Quality Adjusted Life Years; RCT: Randomised Controlled Trial; SCID: Structured Clinical Interview for DSM-VI Disorders; SSQ: Social Support Questionnaire; STAI: State and Trait Anxiety Inventory; TiC-P: Trimbos/iMTA questionnaire for Costs associated with Psychiatric illness; UCL: Utrechtse Coping Lijst.

## Competing interests

The authors declare that they have no competing interests.

## Authors' contributions

CLHB, JO, RPS, PJ, MGP and HB were responsible for the development of the study design and the funding. CLHB was responsible for the development of the intervention and the training of the psychologists. HB is the trial coordinator responsible for the ongoing management of the trial. ADS was responsible for writing the economic evaluation. JLM wrote the draft manuscript. JLM, CB and TV are responsible for the recruitment of participants. All authors have read and approved of the final manuscript.
